# Dung beetles prefer used land over natural greenspace in urban landscape

**DOI:** 10.1038/s41598-022-26841-4

**Published:** 2022-12-23

**Authors:** G. Asha, K. Manoj, T. P. Rajesh, Sangeetha Varma, U. Prashanth Ballullaya, Palatty Allesh Sinu

**Affiliations:** Central University of Kerala, 671316 Kasaragod, Kerala India

**Keywords:** Ecology, Zoology

## Abstract

Urbanization drives land-use and patterns of biodiversity. Yet, very little is known about how biodiversity of structurally different habitats is responded to urbanization. We surveyed coprophagous dung beetles and their ecological functional groups—tunnellers, dwellers, and rollers—in shaded natural semi-evergreen forests of sacred groves and the neighbouring relatively open home gardens of sites that represent three levels of urbanization to address the following questions: (1) Do sacred groves have higher abundance, richness, and diversity of dung beetles than home gardens? (2) Is urbanization a key driver of dung beetle abundance, richness, diversity, and community? (3) Is dung beetle assemblage of sacred groves immune to urbanization? and (4) Which ecological functional groups of dung beetles are affected by urbanization? We hypothesized that the sacred groves have a distinct community, resulting in higher abundance, richness, and diversity of dung beetles than home gardens, and the dung beetle assemblage of sacred groves may be immune to urbanization. We sampled the beetles during wet and dry periods using cow dung as a bait. Against our predictions, dung beetle abundance, richness, and diversity were higher in used lands than sacred groves, particularly in urban landscapes. The two habitats had distinct compositions of dung beetles. Tunnellers and rollers were affected by urbanization, but not dwellers. Heliophilic and synanthropic species characterized by smaller species dominated overall catches in the used lands of urban areas. Results downplay sacred grove as a potential refuge for dung beetles and suggest that the biodiversity of native forests may be affected more by urbanization than the manipulated anthropogenic habitats.

## Introduction

In the Anthropocene, urbanization has been perceived as a major driver of biodiversity change around the world^1,2^. While agriculture-driven land-use change was a threat for biodiversity until early 2000s, increasing human populations and shifting economy from agriculture-based industries to service-oriented industries in developing countries have kept urbanization and urban life style as the reasons for present land-use change. It fragments and transforms the natural and semi-natural areas into a mosaic of altered habitats, which constitutes man-made constructions, parks, and rarely sparsely-distributed urban forests^3,4^. However, increasing emission of greenhouse-gases, pollutants, and dust particles may turn urban forests into the urban heat islands^5^. Therefore, studying the pattern and distribution of biodiversity in urban habitats, particularly the relics of urban forests may inform the likely effect of global warming on biodiversity.

The consequences of expanding urbanization include environmental perturbations, habitat deterioration, local extinction, invasion, and/or even ‘urban’ evolution of species. This can cause reduction/ alteration in the overall biodiversity patterns and ecological functions^2,6–8^. Yet, only recently urban ecosystems and urban biodiversity received attention of conservation biologists and policy makers^7^. Using indicator species, we can discern how communities behave to increasing urbanization. It is suggested that the effect of urbanization may be negative for habitat specialists and larger species, but can be unpredictable for ectotherms, such as insects^9^. Certain bird and mammal species, and ecological communities constituted by them have been reported adapting to urban environments^10–12^. However, studies suggest that urbanization may be associated negatively with the insect biodiversity and the ecological interactions that have insect taxonomic and functional groups as crucial members^13^.

The effect of urbanization on abundance, richness, diversity, and community of invertebrates is inconsistent for various taxa of arthropods^13^. Although the general trend is decreasing with increasing urbanization (reviewed in^14^), social insects such as ants and bees have a positive response to the urbanization^15–21^. However, majority of these studies have come from the first-world countries, which have experienced the urbanization in an intensive fashion for quite a long time before present. Very few studies have examined the effect of urbanization in countries that have exposed to urbanization recently, but in an unprecedented and haphazard manner, such as India.

Among insects, dung beetles are highly diverse across tropics and have been widely considered as bioindicators^22,23^. They extend several crucial ecological functions which include nutrient recycling, secondary seed dispersal, soil aeration, suppression of parasitic flies, among other functions^24,25^. They are good models to study the effect of anthropogenic actions on biodiversity^25^ because they respond to varying degrees of natural and anthropogenic changes in ecosystems^26^, which include forest fragmentation, land use change, reduction in the mammal population, hunting, and defaunation^27^. Also, they are sensitive to subtle changes in the environment due to habitat transformation^27^. Several dung beetle species, either at individual level or at a smaller ecological community level, respond to local habitat characteristics or even the diel period of a day^23,28,29^. Therefore, dung beetles can be a good candidate for studying the effect of urbanization on biodiversity^29^.

How dung beetle species and communities have responded to urbanization has received very less attention or investigated only in certain pockets of the world^24,25,30,31^, but never in Indian cities. Global literature suggests lower species richness and abundance, and higher dominance of certain species in urbanized areas^30,32–35^. The general trend is that dung beetle functional diversity is higher in undisturbed habitats with closed canopy, such as urban forests or greenspaces than disturbed habitats with open canopy, such as settlements and pastures^36–39^. However, Giménez Gomez et al.^40^ reported a greater functional diversity in the disturbed open pasture than the undisturbed native forest. Studies have also suggested that urbanization may drive abundance and richness of various dung beetle functional groups negatively^47,54^. For example, among the nesting guilds, the roller beetles are severely affected by urbanization than the tunnellers and dwellers^29,41^. Among the feeding guilds, the coprophagous beetles are affected severely than necrophagous and generalist species^29^.

In the present study, we used the assemblage of coprophagous dung beetles and their ecological functional groups to address the effect of urbanization in two structurally different habitats—sacred groves and home gardens—in south India. We specifically asked following questions: (1) Do sacred groves have higher abundance, richness, and diversity of dung beetles than home gardens? (2) Is urbanization a key driver of dung beetle abundance, richness, diversity, and community? (3) Is the dung beetle assemblage of sacred groves immune to urbanization? and (4) Which ecological functional groups of dung beetles are affected by urbanization? To address these, we sampled dung beetles from sacred groves (SG) and adjoining home gardens (HG) of an urbanization gradient—low, moderate, and high, in the old-world tropical parts of south India. Sacred groves (described below) are islets of tropical evergreen forests preserved in its original form all over Kerala, parts of Karnataka, Maharashtra, and northeastern states of India. They are conserved by local people for their spiritual values^42,43^ and have a closed canopy. The adjoining home-gardens are the used lands. They have a rather simple habitat structure with relatively open canopy cover. The urbanization level of the sites was assessed by the proportion of built-up area and human population size. Because the sacred groves are miniature forests, our hypotheses are that they have a distinct community, resulting in higher abundance, richness, and diversity of dung beetles than home gardens. Therefore, we also expect that the dung beetle assemblage of at least the sacred groves may be protected from the ill-effects of urbanization. We also expect that the urbanization drive negatively for the abundance of all three ecological functional groups—tunnellers, dwellers, and rollers—in home gardens.

## Materials and methods

### Study sites

**Figure 1:**
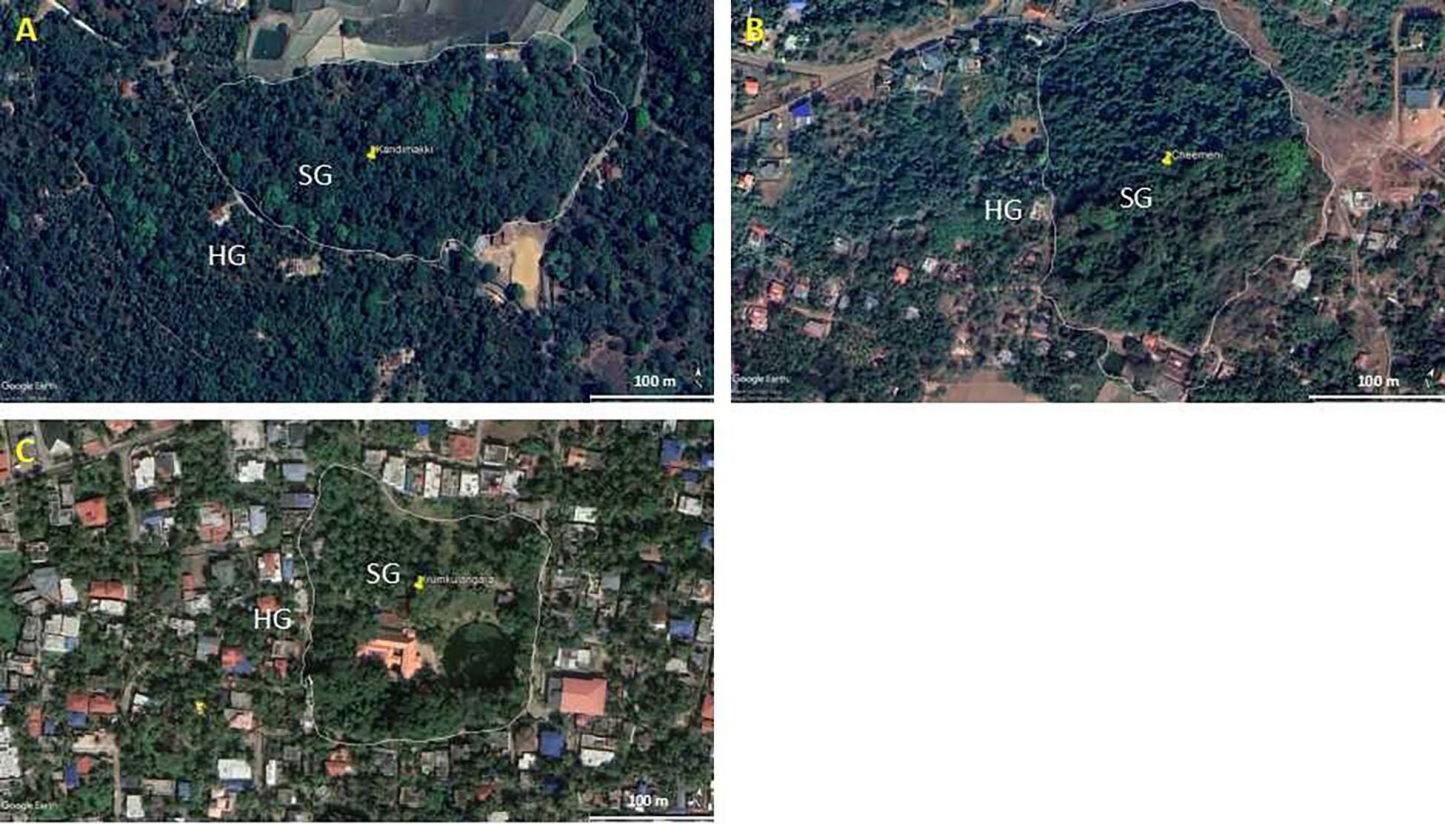
Google earth images of the representative sampling sites in (**A**) less-, (**B**) moderate-, and (**C**) highly-urbanized landscape in Coorg, Kasaragod, and Trivandrum. Copyright: GOOGLE EARTH PRO.

Three locations—Coorg (12°25′0.01ʺN 75°45′0.00ʺE), Kasaragod (12°30′27.5148ʺN 74°59′17.5668ʺE), and Trivandrum (8°31′26.9ʺN 76°56′11.897ʺE)—that represent low (denoted by C.LU), moderate (denoted by K.MU), and high urbanization levels (denoted by T.HU), respectively, were selected for the present study (Fig. 1). They respectively have a human population of 135, 656, and 1509 per sq. km (India Census Report, 2011).

Urbanization was quantitatively assessed by calculating the proportion of the total area under built-up conditions in one-kilometre radius from the sampling sites. We used remote sensing techniques to calculate the proportion of the built-up area. We used data from the Sentinel 2 procured from USGS Earth Explorer (https://earthexplorer.usgs.gov) for the present study. Images of SENTINEL 2A were used for Coorg and Kasaragod and images of SENTINEL 2B were used for Trivandrum. Supervised classification using Maximum Likelihood Classifier was conducted to estimate the built-up area, which was measured on a hectare scale. The minimum and maximum distances between the two locations were 70 km (Coorg and Kasaragod) and 450 km (Kasaragod and Trivandrum). The details of the sampled sites are given in Table S1. The sites in Kasaragod and Trivandrum are located at an altitude below 200 m asl on the boundaries of the Western Ghats biodiversity hotspot; Coorg is located at an altitude above 700 m asl on the Western Ghats.

At each urbanization level (= location), ten independent sites were selected. At each of these sites, two habitats—sacred grove and home garden—were selected on a paired basis, which shared a common boundary. This sampling design reduces the plausible spatial effect that could have been arisen if the habitats of a given site were stood apart, and simultaneously allows us to understand the habitat preference of the beetles.

Sacred groves have the relics of tropical evergreen forests formed through spiritual movement during the British colonial period’s massive logging period centuries ago. For more details of the natural history of the sacred groves, please refer to^42,44,45^. All three locations that fall on three urbanization gradients have 100–200 sacred groves scattered in the mosaic of used lands, residential districts, and business districts. The size of sacred groves varies between two and ten hectares. The sacred groves of all sizes and locations have a moderately thick shade from closed canopy cover and a good litter bed of about 5 cm size, suggesting that the sacred groves maintain structural integrity. Due to agricultural interventions and urbanization, they are located on a mosaic of different agroecosystems and urban centres at present. In all three locations, the sacred grove-home garden pairs were located in the matrix of coffee (Coorg) or coconut orchards (Kasaragod and Trivandrum). Coffee orchards have a structural similarity to the sacred groves, particularly on the canopy cover (Fig. 1A).

### Dung beetle sampling and identification

The sampling was carried out in two seasons—dry (March–April) and wet (July–August)—of 2016. We used fresh cow dung for sampling dung beetles due to restrictions for using other dung in sacred groves. We used two dung piles consisting of 1000 g of cow dung each for sampling beetles from each habitat. Dung piles were laid on bare ground after clearing leaf litter, and kept undisturbed for 24 h. Although our research^46^ showed that guarding pitfall traps around dung piles improve catches of roller dung beetles in protected forests, in sacred groves and home gardens they hardly collected rollers, suggesting that rollers may be less prevalent in fragments and used lands. The dung pile along with one-foot soil were retrieved and soaked in a bucket full of water and allowed the trapped beetles to float on the water. All the beetles were collected, preserved in 90% ethanol, and identified to species using taxonomic keys^47^. The voucher specimens were deposited in the insect collections at the Department of Zoology, Central University of Kerala.

### Data analysis

We examined the sample completeness by running the data of each urbanization level for the sample coverage estimates in iNEXT package^48^. Before we examined the effect of urbanization on dung beetles, we examined whether the home garden type—coffee orchard and coconut orchards—drove richness, abundance, and diversity of dung beetles. We used a generalized linear mixed model to test this; we used sites nested in locations as the random factor and orchard type as the fixed effect. This suggested that Coconut-based homeyards were richer (0.49 ± 0.12, Z = 3.91, P < 0.0005) and abundant (1.89 ± 0.35, Z = 5.5, P < 0.0005) than the Coffee-dominated orchards. However, the two types of home gardens were similar on dung beetle diversity (0.11 ± 0.08, Z = 1.2, P = 0.21). Because the home garden types were different on richness and abundance of dung beetles, home garden type was fitted as a driver in the Model used to study the effect of urbanization on dung beetles of home gardens.

Next, we examined whether the two habitats—home garden and sacred grove—were different on abundance, richness, and diversity of dung beetles in three locations using a set of GLMMs. In these Models, location (Coorg, Kasaragod, and Trivandrum) and habitat (HG and SG) were fitted in interaction terms as the fixed effects. The site was used as a random effect in the models.

In order to study the effect of urbanization the proportion of built-up area in the landscape that comprised of the habitats was fitted as a fixed effect in the models. Because the habitats were different on dung beetle richness, abundance, and diversity in all three locations, we performed our analyses for each habitat type separately. In the final Model, apart from proportion of built-up area, the orchard type of home gardens, area of sacred groves, and altitude were used as other drivers of dung beetle richness, abundance, and diversity.

The site was considered as an independent replicate in all analyses. Therefore, the dung beetles of dung and season replicates of each site were pooled to obtain a single site × species matrix for each habitat. All the analyses were performed using R and R-packages lme4^49^, car^50^, MuMIn^51^, and vegan^52^. Abundance of overall dung beetles and ecological functional guilds, richness, Shannon diversity, and species composition were our response variables in the models. For the models having abundance and richness as response variables, negative binomial distribution was fitted as the error family. For the models having Shannon diversity as the response variable, Gaussian distribution was fitted as the error family. The residuals were checked for the normality for all fitted models using the R-package, DHARMa^53^. The significance of the overall models was tested using Anova function available in the R-package car^50^. The regression value—marginal and complete—of the models was tested using the R-package, MuMin^51^.

To examine the changes in community composition of dung beetles among urbanization levels, Permutational Multivariate Analyses of Variance (PERMANOVA) on the similarity matrices of species abundance (Bray–Curtis dissimilarity index) were performed using the ‘adonis’ function of R-package vegan^52^. We used ‘betadisper’ function available in ‘vegan’^52^ to test whether the locations at three urbanization levels were homogeneously dispersed in relation to their species. The results were graphically illustrated in multi-dimensional scaling (PCoA). Dissimilarity of two habitats on dung beetle composition was checked using ANOSIM available in ‘vegan’ package. Bray–Curtis index was used as a measure of dissimilarity index. Heatmap was generated using the R-package ‘gplots’^54^ to identify species communities and to illustrate similarity among locations on species composition. All analyses were performed using the program R 4.1.0^55^.

## Results

### Patterns of dung beetle abundance and richness

**Figure 2:**
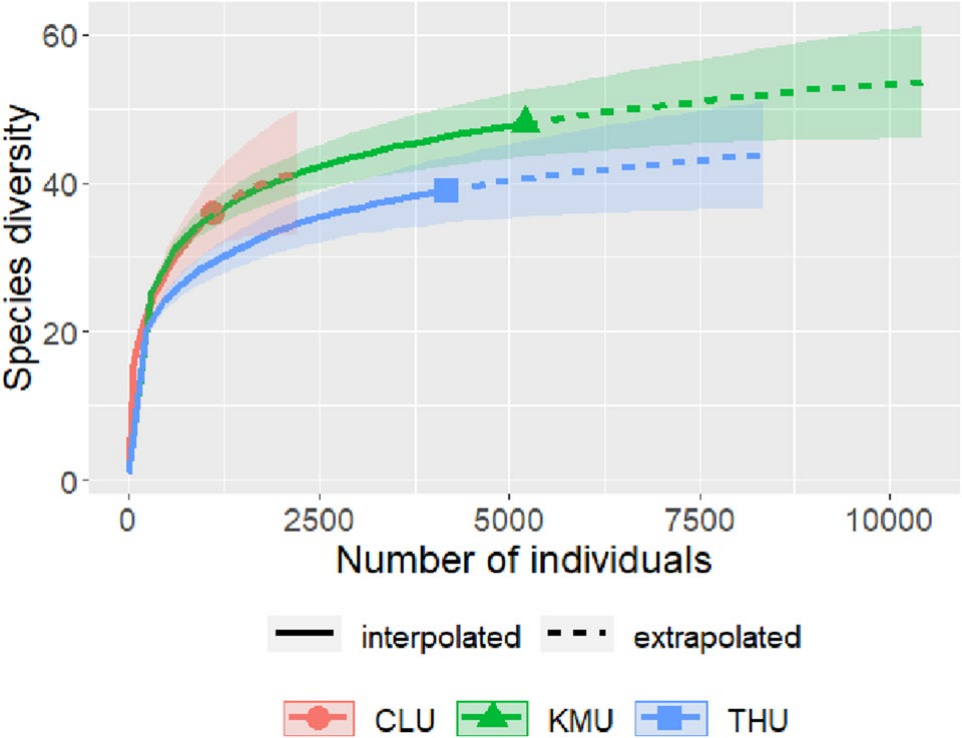
Sample coverage based rarefaction curve of dung beetles in less-, moderate-, and highly-urbanized landscape. The shaded areas indicate a 95% confidence interval.

We collected a total of 10,417 individuals representing 11 genera and 72 species of dung beetles from the study (Table S2). Out of the 72 species, ten, sixteen, and nine species were collected exclusively from less urbanized (Coorg), moderately urbanized (Kasaragod) and highly urbanized sites (Trivandrum), respectively. Sixteen species were common to sites of all three locations. The estimated sample coverage was 99.13%, 99.85% and 99.83% for the less-, moderately-, and highly-urbanized locations, respectively (Fig. 2). A total of 1035 (103.5 ± 72.16) individuals belonging to 35 (13.7 ± 4.13) species, 5211 (521.1 ± 613.04) individuals belonging to 48 (19.9 ± 5.25) species, and 4171 (417.1 ± 386.62) individuals belonging to 39 (17.1 ± 4.74) species were respectively collected from less-, moderately-, and highly-urbanized landscapes.

**Figure 3:**
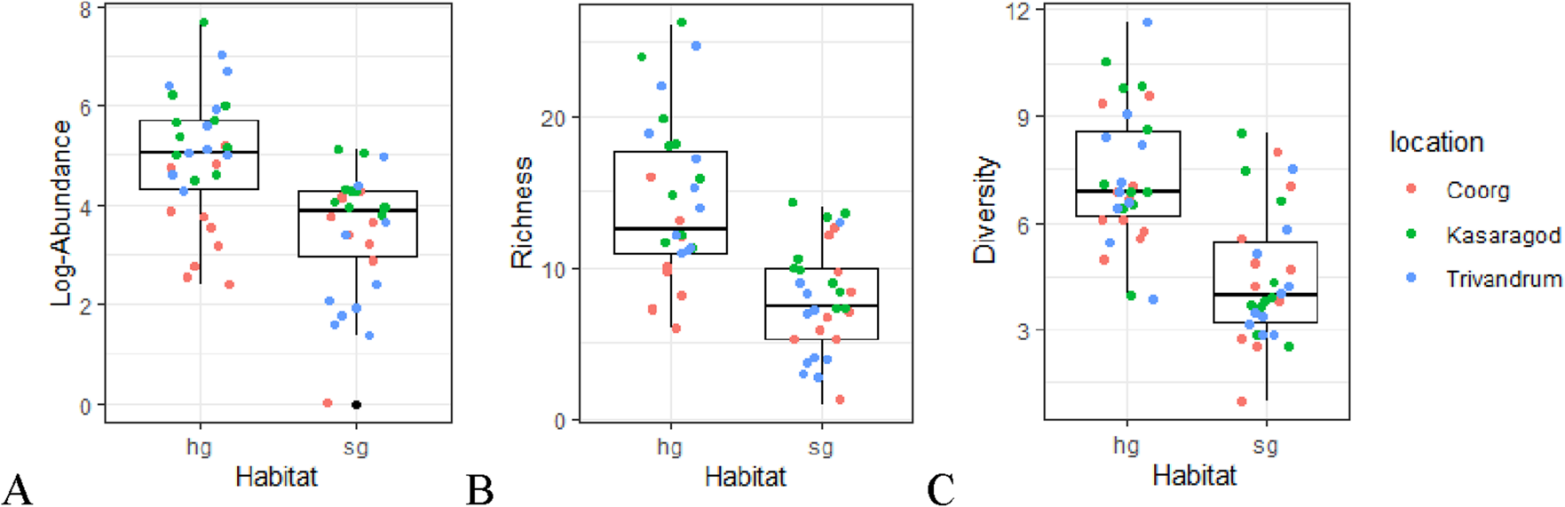
In pooled locations, dung beetle abundance, richness, and diversity were higher in home gardens than sacred groves. The sites of three locations are jittered on the box plots.

Sacred groves had a considerably lower abundance (− 1.45 ± 0.18, Z = − 8.07, P < 0.0005), richness (− 0.71 ± 0.07, Z = − 10.27, P < 0.0005), and Shannon diversity of dung beetles (− 0.57 ± 0.08, Z = − 6.8, P < 0.0005) than home gardens (Fig. 3). This pattern was maintained consistent for all the locations, but less-urbanized Coorg (Table S3), where dung beetles were collected by similar numbers in home gardens and sacred groves (− 0.14 ± 0.19, Z = − 0.73, P = 0.43); however, richness was marginally lower in sacred groves (− 0.26 ± 0.13, Z = − 2.03, P = 0.045) (Table S3). The interaction between location and habitat type drove abundance, richness, and Shannon diversity of dung beetles (Table 1).

**Table 1:** Results of generalized linear mixed effect models for dung beetle abundance, richness, and diversity.

	χ^2^	DF	P-value	R^2^_GLMM(M)_	R^2^_GLMM(C)_
**Abundance**				**0.52**	**0.76**
Location (L)	14.4	2	< 0.0005		
Habitat (H)	101.9	1	< 0.0005		
L*H	54.9	2	< 0.0005		
**Richness**				**0.49**	**0.49**
Location	8.5	2	0.01		
Habitat	96.6	1	< 0.0005		
L*H	23.7	2	< 0.0005		
**Shannon diversity**				**0.31**	**0.36**
Location	0.62	2	0.73		
Habitat	49.04	1	< 0.0005		
L*H	8.14	2	0.02		

The effect types are location, habitat, and an interaction between location and habitat. R^2^ marginal denotes the proportion of variance explained by the fixed effect; R^2^ conditional denotes the proportion of variance explained by both the fixed and random effects.

### Effect of urbanization

#### Sacred groves

In sacred groves, richness decreased with the proportion of built-up area (− 2.18 ± 0.66, Z = − 3.31, P = 0.005), but increased when the neighbouring used land was Coconut orchards (0.73 ± 0.31, Z = 2.31, P = 0.02). Altitude (0.14 ± 0.09, Z = 1.54, P = 0.12) and area of sacred groves (− 0.06 ± 0.05, − 1.09, P = 0.28) were poor drivers of dung beetle richness. The Model explained 41% of variation. Abundance also decreased with the proportion of built-up area (− 5.1 ± 1.07, Z = − 2.98, P = 0.002). Altitude (− 0.28 ± 0.23, Z = − 1.18, P = 0.23), the orchard type in used land (− 0.24 ± 0.92, Z = − 0.26, P = 0.7), and area of sacred groves (− 0.13 ± 0.12, Z = − 1.03, P = 0.3) were not driving abundance of dung beetles in sacred groves. The model explained 35% of the variation in the data. The diversity of dung beetles also decreased with the proportion of built-up area in the landscape (− 0.87 ± 0.31, Z = − 2.8, P = 0.005). However, the dung beetle diversity in sacred groves increased with the altitude (0.30 ± 0.05, Z = 5.6, P < 0.0005) and when the used land surrounding sacred groves was Coconut orchards (0.89 ± 0.21, Z = 4.32, P < 0.0005). The area of sacred groves was unimportant for the diversity too (− 0.04 ± 0.06, Z = 0.7, P = 0.48). The model explained 19% of total variation in the diversity of dung beetles.

#### Home gardens

In home gardens, richness (− 0.8 ± 0.4, Z = − 1.96, P = 0.049) and abundance (− 2.5 ± 1.02, Z = − 2.43, P = 0.01) decreased with the proportion of built-up area in the landscape, but diversity was unaffected by the proportion of built-up area (0.12 ± 0.17, Z = 0.65, P = 0.51). As mentioned before, the Coconut based orchards had remarkably more abundance (2.16 ± 0.61, Z = 3.57, P < 0.0003) and marginally higher richness of dung beetles (0.41 ± 0.07, Z = 1.7, P = 0.09) than Coffee-based orchards. The diversity was unaffected by the orchard type of home gardens. The altitude and area of sacred groves were less crucial drivers of abundance, richness, and diversity of dung beetles. The fixed variables predicted 57%, 44%, and 3% of total variation in abundance, richness, and diversity of dung beetles.

#### Functional groups

Overall, tunnelling dung beetles were abundant than dwellers in the whole study (GLM.NB: 0.81 ± 0.32, Z = 2.54, P = 0.01). The dwellers were common in the habitats of all three locations. However, rollers were collected very less in the whole study (N = 17), and were a total miss in the samples of highly-urbanized landscape. The abundance of tunnellers was driven by the interaction between location and habitat (Anova: X^2^ = 50.56, DF = 2, P < 0.00005). The dwellers’ abundance was not driven by the interaction between habitat and location (Anova: X^2^ = 4.92, DF = 2, P = 0.08).

Overall, the abundance of dwellers (− 4.39 ± 2.02, Z = − 2.17, P = 0.02) and tunnellers (− 2.69 ± 1.09, Z = − 2.45, P = 0.01) were negatively associated with the proportion of built-up area. The dweller abundance of home gardens (− 4.21 ± 2.3, Z = − 1.83, P = 0.06) and sacred groves (− 8.14 ± 4.2, Z = − 1.94, P = 0.05) decreased marginally, but tunneller abundance decreased considerably both in sacred groves (− 4.71 ± 1.70, Z = − 2.77, P = 0.005) and home gardens (− 2.26 ± 1.08, Z = − 2.08, P = 0.03) with the proportion of built-up area.

#### Species composition and habitat associations

**Figure 4:**
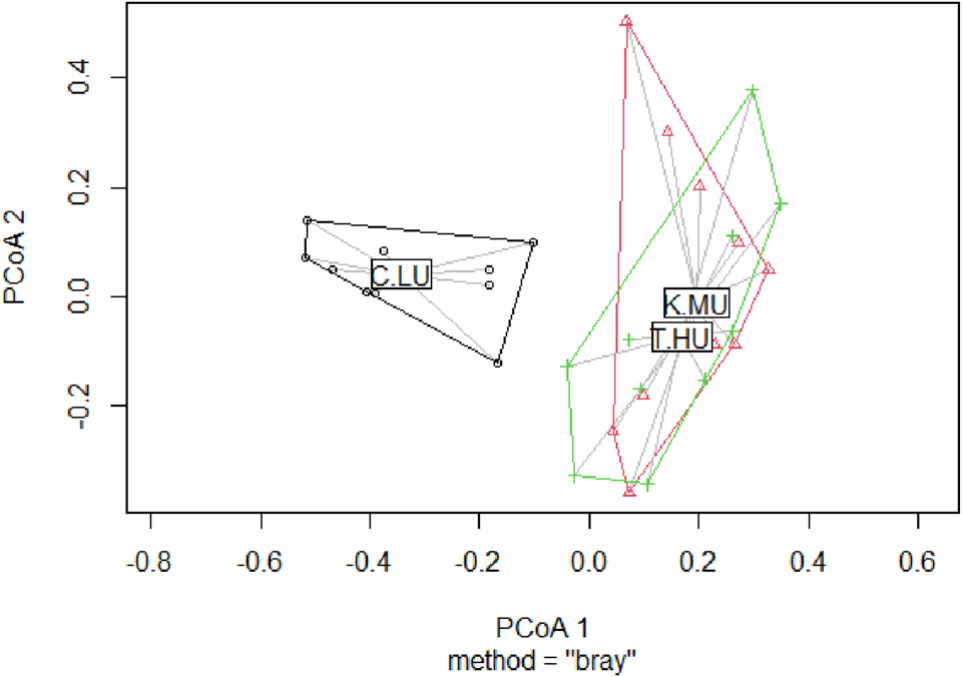
The MDS plot ordinates sites on community composition of dung beetles in the less urbanized (C.LU), moderately urbanized (K.MU) and highly urbanized (T.HU) landscapes.

The dung beetle community of the urbanized sites was characterized by the dominance of a subset of dung beetle species constituted by *Tiniocellus spinipes* Roth, 1851 (N = 2125; 118.05 ± 259.74), *Onthophagus turbatus* Walker 1858 (N = 1460; 48.66 ± 56.89), *Onthophagus favrei* Boucomont 1914 (N = 1248; 41.6 ± 50.35), *Tibiodrepanus setosus* Wiedemann 1823 (N = 1019; 37.77 ± 124.66), *Onthophagus cervus* Fabricius 1798 (N = 901; 37.54 ± 52.56), and *Caccobius meridionalis* Boucomont 1914 (N = 797; 31.88 ± 62.61). The abundance of these species was much higher in the home gardens than sacred groves (Table S2). Most of these common dung beetles were smaller in size. The dung beetle communities of moderate and highly-urbanized sites were similar, but were different from the community of less-urbanized sites (PERMANOVA: Pseudo-F = 4.53, R^2^ = 0.25, p = 0.01) (Fig. S2). The group dispersion was not significant (ANOVA: F = 0.16, P = 0.84) (Fig. 4). The average distance to the median were 0.44 (C.LU), 0.41 (K.LU) and 0.43 (T.HU) (Fig. S1). The habitats were dissimilar on the composition of dung beetles in less (ANOSIM R = 0.32, P = 0.001), moderate (R = 0.39, P = 0.001), and highly urbanized landscapes (ANOSIM R = 0.39, P = 0.001).

## Discussion

The present study compared the coprophagous dung beetle assemblages between two structurally different habitats—the sacred groves and home gardens—in sites of three levels of urbanization in south India. We expected that the sacred groves, owing to their structural integrity of a tropical semi-evergreen forest, have a better assemblage of dung beetles than the home gardens, and at least the assemblage of sacred groves may be immune to urbanization. Against our predictions, the results show that (a) the sacred groves in general have less richness, abundance, and diversity than the home gardens, and have a community dissimilar to home gardens, and (b) urbanization is negatively associated to abundance, richness, diversity, and composition of dung beetles in both the habitats; the rollers and tunnellers are affected more than the dwellers.

Our findings downplay the importance of sacred groves in conserving dung beetles at least in more urbanized landscapes, and partially disagree to the general perception that the greenspace in an urban matrix can provide refuge for dung beetles^25,29,35^. Our sampling design allows the beetles to choose their habitat, yet majority of them preferred the dung resources of used lands. The lower richness of dung beetles in sacred groves of all three urbanization levels confirms that it is not a local pattern restricted to one or two sites, but a trend seems typical for South India as the present study covered thirty sacred grove-home garden pairs at a greater geographical scale.

Although the locations of three urbanization levels stood apart greatly, a good number of the dominant species was common to all three landscapes, justifying the validity of the experimental design. Precisely, five of the ten most dominant species that contribute to 82% (C.LU) to 90% (K.MU & T.HU) of the overall abundance were collected from the habitats of all three urbanization levels (Table S2). They are *Onthophagus turbatus*, *O. favrei*, *O. fasciatus* Boucomont, 1914, *Caccobius meridionalis*, and *Tibiodrepanus setosus*. This result reveals the plasticity of these species to adapt to urban environment. However, all these species have consistently higher abundance in home gardens than sacred groves in all three locations, including the less-urbanized Coorg. It is also likely that these species may prefer disturbed habitats or habitats with cleared canopy cover.

The sites of all three urbanization levels have their own share of unique species. Twenty-three percent of the species of highly-urbanized Trivandrum is unique to it; for the less- and moderately-urbanized locations, the shares of unique species are 28% and 33%, respectively. The most-dominant species of moderately- and highly-urbanized locations—*Tiniocellus spinipes*—was a total miss in both the habitats of less-urbanized landscape. Our literature survey reveals that it is a synanthropic—adapted to live in urban environment—species^56^. Interestingly, about 90% of the total catches of this species in more urbanized landscapes were collected from the home gardens, which suggests that it is a heliophilic—adapted to the lands of cleared canopy—species too. *Tiniocellus spinipes* is a dweller, therefore the substratum may not affect their use of dung resources. The results agree that the urbanization can alter the community of dung beetles^56^.

The less-urbanized Coorg also has its own set of unique species. Its dung beetle community is also different from the community of the other two urbanization levels. Excluding the five commonly collected species, majority of the species collected here are asynanthropic and heliophobic. Coorg, among the three locations, has many species collected abundantly in sacred groves than the home garden, a pattern scarcely seen for more urbanized locations. They include *O. quadridentatus* Fabricius, 1798, *O. ensifer* Boucomont, 1914, *O. vladimiri* Frey, 1957, *Copris carinicus* Gillet, 1910, *C. sodalis* Walker, 1858, and *Copris signatus* Walker, 1858. All of them are tunnellers and specialists of non-cattle dung, such as elephant and wild boar^56–58^. Coorg has many nature reserves and the used lands are dominated by Coffee, a system known to support biodiversity^44^. However, because of tourism, Coorg is also developing, and the presence of the five common heliophilic and synanthropic species mentioned above may be indicating the changing pattern of dung beetle community and the growing disturbance in Coorg. It is bothersome and worth analysing the trend of these five common species in the overall landscape of Coorg to identify the spots that need conservation and proper management. The results vouch the indicator potential of dung beetle species.

Although our expectation was that the sacred groves, owing to their possession of forests and closed canopy, have more species and abundance of dung beetles than the home gardens, we witnessed a reverse pattern with the home gardens conserving more species and abundance than the sacred groves, a pattern reported not rarely^23,59–61^. While this pattern was very clear in moderately- and highly-urbanized landscapes, in less-urbanized location, the abundance was similar for both the habitats, and richness was marginally higher in sacred groves. The natural source of dung in urban landscapes are cats, dogs, and livestock animals including chicken^24,29,35^. The moderately- and highly-urbanized sites of the present study also have them as the source of dung, but in both the habitats of less urbanized location, free-ranging cattle, wild boars, civets, and primates also supply dung. This difference in the natural source of dung is likely in urban gradient and rural–urban dung beetle diversity studies. This could also be a reason why the sacred groves and home gardens of less-urbanized landscape had similar abundance and richness of dung beetles. However, although Coffee agroforests have been known for biodiversity conservation in tropics, the results suggest that the dung beetles may be richer and abundant in canopy-cleared habitats than the closed habitats. When Coffee had 26 species of dung beetles, the Coconut-based orchards had 46 (Kasaragod) and 36 (Trivandrum) species. However, in agreement to our results, Giménez Gómez et al.^40^ found that open pasture had higher functional diversity of dung beetles than native forests, and attributed this pattern to the physiological traits of dung beetle species. It seems the thermal tolerance of dung beetle species collected in the present study could also be a reason for high richness of dung beetles in Coconut orchards^62^. Although it was not a main topic of the present research, the findings that the dung beetle richness of sacred groves is influenced by the quality of the used lands around them need a critical analysis, and may be a potential topic for future research.

Overall, the three landscapes that differed by the level of urbanization have collected statistically similar richness of dung beetles—35 (C.LU), 48 (K.MU), and 38 (T.HU), but a very different number of individuals—1035 (C.LU), 5211 (K.MU), and 4171 (T.HU). Although our first expectation was that the possible different species community might explain this contradictory pattern between species richness and abundance, that did not hold true. As discussed above, five dominant species in the list of first ten dominant species were common for all three locations. Most of these species, because are synanthropic and heliophilic, have been collected more in the moderately and highly-urbanized locations.

Dung beetle ecological functional groups respond to urbanization differently^29^. Tunnellers, followed by dwellers and rollers is a typical pattern suggested for the abundance of dung beetles in tropics^63^ and the Western Ghats biodiversity hotspots^64^. Our findings agree to this. Like overall abundance, the abundance of some of these functional groups also decreased with the proportion of built-up area in both the habitats. Tunnellers are important functional groups of dung beetles^65^, which perform the functions of fixing dung in soil and aerate the soil. The interaction between location and habitat drives the abundance of tunnellers but not dwellers. Rollers are the other important functional group of dung beetles adversely affected by urbanization^29,41,66^. Rollers are collected by very few numbers in the present study, and are not collected at all in the highly-urbanized landscape, suggesting that urbanization could be a potential threat for the rollers. As rollers have the least reproductive potential and high sensitivity to landuse change, among all dung beetle functional guilds they are highly vulnerable to urbanization^67^.

Another implication of urbanization is the poor representation of larger dung beetles or decreased biomass in urbanized areas^29,68^. The present study rarely collected the largest dung beetles reported for the Western Ghats biodiversity hotspot, such as *Catharsius* Hope, 1837 and *Heliocopris* Hope, 1837 (23–37 cm). The lone dung beetle of this band collected in the present study was *Catharsius molossus*, and that was collected from the home garden of moderately-urbanized landscape. Several medium-sized dung beetles belonged to tunneller guild, such as *Copris* spp (16–17.5 mm), were collected from both the habitats of all three landscapes. However, they were collected more in sacred groves than home gardens in less urbanized landscape. In moderate and highly-urbanized landscape, they were collected by similar numbers in both the habitats and by lesser number in sacred groves than home garden, respectively. Dominance of smaller species is predicted for dung beetles in disturbed areas^35,56,68^. Our results agree to these projections. *Tiniocellus spinipes*—a small species—is the most dominant species collected in moderately- and highly-urbanized landscapes, however, it is not collected in less-urbanized landscapes. This despite is intriguing, could be also driven by the species community in ephemeral dung, and need further investigations.

## Conclusion

Urbanization is an inevitable anthropogenic process. Increasing air pollution, local warming, and deteriorating human health and mental health prompted cities valuing urban forests and urban biodiversity. Diversity of functional groups, such as dung beetles is essential for maintaining a healthy urban ecosystem. The sites of all three urbanization levels we considered in the present investigation have natural forest relics, the sacred groves. In short, the present study suggests that the urbanization could be a potential driver of dung beetles in urban forests rather than the used lands. Urbanization could also negatively affect the community structure of dung beetles of certain functional guilds, such as rollers and larger dung beetles, a pattern observed by other studies^24,28,29^. The results suggest that the biodiversity of natural forests may be affected more by urbanization than the used anthropogenic lands. A knowledge of how key elements of insect diversity and functions they render to ecosystems behave to urbanization is a key to our understanding of how biodiversity might behave to global warming as cities offer a heat island^5^.

## Supplementary Information

Supplementary Information 1.

Supplementary Information 2.

## Data Availability

All data generated or analysed during this study are included in this published article (and its Supplementary Information files).
