# Supplementary material for: Dung beetles prefer used land over natural greenspace in urban landscape
Source: Sci Rep. 2022 Dec 23;12:22179. doi: 10.1038/s41598-022-26841-4 (PMC9789146; doi:10.1038/s41598-022-26841-4)
Supplement: Supplementary file 1 — Supplementary Information 1. [file 41598_2022_26841_MOESM1_ESM.docx]

**Dung beetles prefer used land over natural greenspace in urban landscape**

**G. Asha*, K. Manoj, T.P. Rajesh, Sangeetha Varma, Prashanth Ballullaya U, Palatty Allesh Sinu***

**Central University of Kerala 671316, Kerala, India**

**ashagkoppal@gmail.com;** [**sinu@cukerala.ac.in**](mailto:sinu@cukerala.ac.in)

**Electronic Supplementary Materials**

**Supplementary Table S1** Detailed of the sampling sites in three urbanization levels (C.LU: less urbanized Coorg, K.MU: moderately urbanized Kasaragod and T.HU: highly urbanizedTrivandrum)

| Urbanization level | Location | Site name |
| --- | --- | --- |
| C.LU | Kutta-Badaga | Kappangadi Ayyappa Devarakadu |
| C.LU | Palangala | Palangala Keemani Ayyappa Devasthana |
| C.LU | Bettoli | Bettoli Shree Bhadrakali Devasthana |
| C.LU | Heggala | Heggala Ayyappa Bhagavathi Devasthana |
| C.LU | Kottoli | Kottoli Shree Dhara Maheshwara Devasthana |
| C.LU | Kolathod | Kolathodu Ayyappa Subrahmanya Devasthana |
| C.LU | Kandimakki | Kandimakki Thrimoorthi Devasthana Balagodu |
| C.LU | Kunjila | Ayangeri Ayyappa Devarakadu |
| C.LU | Nariyandada | Nariyandada Bhagavathi Devasthana |
| C.LU | Porad | Shree Ponnya Bhagavathi devasthana |
| K.MU | Muthappanarkavu | Muthappanarkavu |
| K.MU | Cheemeni | Cheemeni 1 |
| K.MU | Cheemeni | Cheemeni 2 |
| K.MU | Kammadam | Kammadam 1 |
| K.MU | Kammadam | Kammadam 2 |
| K.MU | Kammadam | Kammadam 3 |
| K.MU | Nileshwar | Puthiyaparambathkavu |
| K.MU | Nileshwar | Cheermakavu |
| K.MU | Nileshwar | Mannampurathkavu |
| K.MU | Valiyaparamba | Idayilakkadu |
| T.HU | Pachallur | Chokkankavu |
| T.HU | Manacaud | Irumkulangara Bhagavathi temple |
| T.HU | Kattakada | Karingal Thottikkara Nagarukavu |
| T.HU | Chaavarkodu | Trikkunnathukavu |
| T.HU | Vattiyurkavu | Arappura rewSaraswathiyamman |
| T.HU | Kattakada | Veeranakavu |
| T.HU | Kollamkkodu | Moopuramkavu |
| T.HU | Irinjayam | Irinjayamkavu |
| T.HU | Uchakkada | Ooruvilakamkavu |
| T.HU | Chittayikodu | Valiyakavu |

**Supplementary Table S3** Parameter estimates (Z-values (β±SE)) of generalized linear mixed effect models testing responses for abundance, richness, and diversity of dung beetles towards habitats; the parameters given in table are for sacred groves over home gardens. *indicates p<0.05; † indicates p<0.01

| Landscape | Abundance | Richness | Shannon |
| --- | --- | --- | --- |
| Overall | -8.07 (-1.45±0.18)† | -10.27 (-0.71±0.07)† | -6.8 (-0.57±0.08)† |
| C.LU | -0.73 (-0.14±0.19) | -2.03(-0.26±0.13)* | -2.87 (-0.34±0.12)* |
| K.MU | -4.85 (-1.45±0.3)† | -6.04 (-0.66±0.11)† | -3.21 (-0.47±0.15)† |
| T.HU | -13.6 (-2.71±0.19)† | -9.01 (-1.12±0.12)† | -6.42 (-0.89±0.14)† |


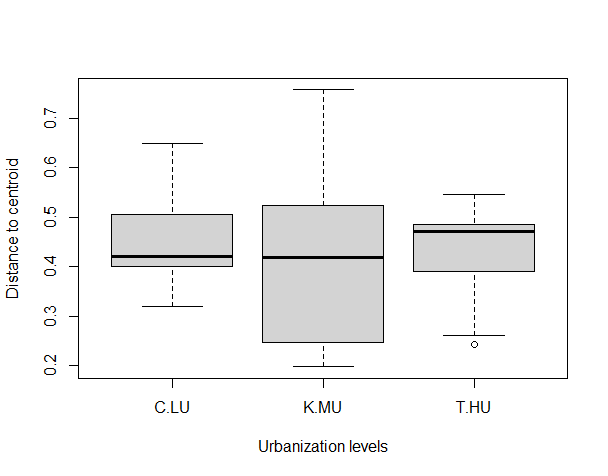


**Supplementary Fig. S1** Box plot shows dispersion in three urbanization levels


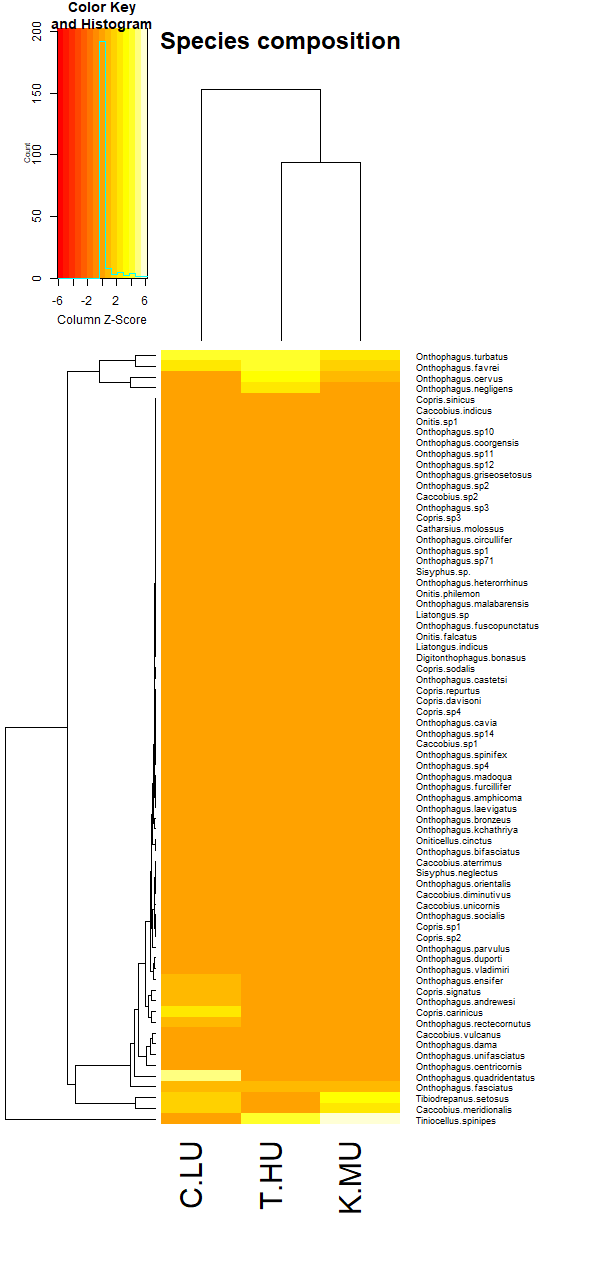


**Supplementary Fig. S2** Heat map of species distribution and abundances of dung beetle collected in less urbanized (C.LU), moderately urbanized (K.MU) and highly urbanized (T.HU) landscapes.
